# Construction of a prediction model for Alzheimer’s disease using an AI-driven eye-tracking task on mobile devices

**DOI:** 10.1007/s40520-024-02882-9

**Published:** 2024-12-27

**Authors:** Qinjie Li, Jiaxin Yan, Jianfeng Ye, Hao Lv, Xiaochen Zhang, Zhilan Tu, Yunxia Li, Qihao Guo

**Affiliations:** 1 Department of Gerontology, Shanghai Jiao Tong University Affiliated Sixth People’s Hospital, No. 600, Yi Shan Road, Shanghai, 200233 China; 2 Department of Neurology, Tongji Hospital, School of Medicine, Tongji University, Shanghai, China; 3 Department of Neurology, Shanghai Pudong Hospital, Fudan University Pudong Medical Center, 2800 Gongwei Road, Shanghai, 201399 China; 4 NeuroWeave, Co., Ltd, Shanghai, China; 5 Shanghai Key Laboratory of Vascular Lesions Regulation and Remodeling, Shanghai, China

**Keywords:** Alzheimer’s disease, Eye movement, Eye-tracking, Machine learning

## Abstract

**Background:**

Eye-movement can reflect cognition and provide information on the neurodegeneration, such as Alzheimer’s disease (AD). The high cost and limited accessibility of eye-movement recordings have hindered their use in clinics.

**Aims:**

We aim to develop an AI-driven eye-tracking tool for assessing AD using mobile devices with embedded cameras.

**Methods:**

166 AD patients and 107 normal controls (NC) were enrolled. The subjects completed eye-movement tasks on a pad. We compared the demographics and clinical features of two groups. The eye-movement features were selected using least absolute shrinkage and selection operator (LASSO). Logistic regression (LR) model was trained to classify AD and NC, and its performance was evaluated. A nomogram was established to predict AD.

**Results:**

In training set, the model showed a good area under curve (AUC) of 0.85 for identifying AD from NC, with a sensitivity of 71%, specificity of 84%, positive predictive value of 0.87, and negative predictive value of 0.65. The validation of the model also yielded a favorable discriminatory ability with the AUC of 0.91, sensitivity, specificity, positive predictive value, and negative predictive value of 82%, 91%, 0.93, and 0.77 to identify AD patients from NC.

**Discussion and Conclusions:**

This novel AI-driven eye-tracking technology has the potential to reliably identify differences in eye-movement abnormalities in AD. The model shows excellent diagnostic performance in identifying AD based on the current data collected. The use of mobile devices makes it accessible for AD patients to complete tasks in primary clinical settings or follow up at home.

## Introduction

Alzheimer’s disease (AD) is a neurodegenerative disease that mainly occurs in the old people and is the most common cause of dementia [1–2]. The current estimated global prevalence of AD ranges between 33 and 38.5 million individuals [3]. China has 249·49 million people aged above 60 years, suggesting a high prevalence of AD [4]. According to the National Institute of Aging–Alzheimer’s Association (NIA–AA) workgroup, neuropathologic examination for amyloid-beta burden and neurofibrillary tau deposits is the gold standard for defining AD [2, 5]. The current biomarkers tests used for AD are either expensive or invasive. Timely screening or monitoring neuropathological changes of AD with affordable and noninvasive markers is a crucial healthcare priority in clinical settings.

The progression of the AD leads to cognitive impairments in memory, language, and executive function, ultimately resulting in a gradual deterioration of activities of daily living [1]. Those clinical symptoms are also accompanied by altered eye movement. The eye movement information can indirectly link cognitive states through the similar structural or functional changes in the cortical regions in AD [6–7]. The pathways of visual information initially processed in the visual cortex and conveyed to the parietal and frontal lobes [6–7], which were also found to be impacted in AD [8]. The fusiform face area in the medial temporal lobe, located in the fusiform gyrus, which is responsible for facial identification, is affected by AD pathology [9]. It has been reported that eye movements could be sensitive to the severity of dementia and potentially serve as a useful biomarker for AD [10–12].

The clinical common assessment of eye movement includes fixate, smooth pursuit, pupillary responses, and saccades. Based on the fundamental eye movements, more complex physiological tasks are derived, such as visual search, scene exploration, antisaccade (AS) task, and visual paired comparisons (VPC) [6, 10, 13]. Antisaccades are the fast movements of the eyes shift gaze from one spatial location to another in the opposite direction of a target. Research suggests that AD-related anti-saccade changes may reflect impaired inhibitory control [14]. Previous studies have reported that patients with AD exhibit a higher frequency of incorrect saccades towards the target and demonstrate increased latency when performing anti-saccades and making corrective saccades following an error compared to healthy controls [6, 10, 13]. In the VPC task, participants are first presented with a visual stimulus for a fixed period of time; then, they are presented with a pair of stimuli, and eye-tracking information is recorded by comparing the time spent on novel and familiar images. The VPC tasks are sensitive to memory impairment [15], one previous study has confirmed that VPC performance can effectively evaluate visual memory recognition in individuals with AD and normal cognition, and accurately distinguishing those with good sensitivity [16].

Traditionally, eye tracking data is captured using a high frame rate camera in a commercial-grade eye tracker capable of capturing various visual features [13, 15, 17]. These data are then analyzed either with manufacturer-provided software or manually inspected by researchers experienced in evaluating eye tracking metrics. However, the high cost and expenses for maintenance of commercial-grade eye trackers often limits their accessibility to research institutions rather than everyday clinical settings [18]. Currently, the passive monitoring of daily activity via mobile device provides portable methods of tracking behavioral changes over time. Compared with traditional eye-tracking cameras, embedded cameras in mobile devices represent a cheap and easily accessible option for eye-tracking. The combination of phone cameras and machine learning algorithms could facilitate the development of a more rapid and economical method for cognitive assessment on a large scale [19–20]. Recently, research has introduced a concept for the utilization of embedded web cameras in neuropsychological and neuropsychiatric assessments, demonstrating their validity as an alternative to traditional eye-tracking equipment for VPC tasks [18, 21]. However, further validation against clinical-grade eye trackers remains necessary.

To investigate this, we utilized an Eye-Tracking Neurological Assessment (ETNA™) based on mobile devices to collect antisaccades and VPC data and used machine-learning algorithms to generate models. In contrast to conventional eye trackers, this technology employs an AI-based eye-tracking technique, facilitating reliable and accurate tracking of eye movements through the embedded camera on the pad or tablet. This technique first identifies facial landmarks within the video, including head and eye orientation [22–23]. By establishing a mapping relationship between the identified facial features and the gaze points on the screen, an eye movement attention prediction model is constructed. A similar technology has also been employed in research pertaining to Parkinson’s disease [24]. In this paper, we employ a series of visual processing and anti-saccade tasks conducted within 6 min using mobile devices to collect data. Subsequently, we develop a machine learning model and nomogram to distinguish between individuals with AD and healthy controls and predict the probability risk of AD.

## Methods

### Participants

A total of 273 participants (61.2% females; Mean age = 70.46 years; range: 50–90 years) were recruited from the Department of Geriatrics, Shanghai Sixth People’s Hospital, the Department of Neurology, Shanghai Tong Ji Hospital, and the community between April 2021 to July 2023. Participants with history of head trauma, alcoholism, drug abuse, or other neuropsychiatric disorders that could potentially impact cognitive function, such as depression, anxiety, were excluded. In addition, participants with eye disorders that could potentially affect the eye move examinations were excluded from the study. The study received approval from the ethics committee of the Shanghai Sixth People’s Hospital or Shanghai Tong Ji Hospital, and all participants provided informed consent.

All participants were subdivided into 107 normal controls (NC) and 166 AD. Normal controls were recruited via the community with a normal performance on standardized cognitive tests that did not meet the criteria of MCI and dementia, meanwhile, had no subjective cognitive complaints by themselves and verified by informants. AD was diagnosed based on the National Institute of Aging and Alzheimer’s Association (NIA-AA) criteria [25]: clinically identified dementia, which was recorded by mini-mental state examination and confirmed by a neuropsychological test; progressive deterioration of memory and other cognitive functions; deficits in 2 or more domains of cognition; insidious onset of symptoms and a history of cognitive decline by observation.

### Neuropsychological tests

All participants underwent a battery of comprehensive neuropsychological assessments. The Mini-Mental State Examination (MMSE) and the Chinese version of Montreal Cognitive Assessment Basic Version (MoCA-B) were applied in the general cognitive screening tests [26–27]. Episodic memory function was evaluated with the Auditory Verbal Learning Test-Huashan [28], including long-term delayed recall and recognition. Attention/executive function was evaluated with the Shape Trails Test Parts A and B [29]. Language function was evaluated with the Boston Naming Test and Animal Verbal Fluency Test [30–31]. Neuropsychological tests were conducted by trained raters who were blinded to the diagnosis.

### Eye movements task

#### Apparatus

The testing hardware consisted of a Xiaomi Mi 5 Pro Android tablet with a screen size of 12.4 inches, a screen resolution of 1280*800 pixels, and a refresh rate set to 60 Hz. The front-facing camera on the tablet had a sample rate of 30 Hz. The testing software was developed by the NeuroWeave, Co., Ltd. team and was compatible with Android 8.0 and above. Once the task was initiated, participants were recorded using the tablet’s front-facing camera, and the recorded video was streamed to our Alibaba Cloud server instance for further analysis. An AI-based eye-tracking model downloaded the video to the local server for analysis, and the results were then sent back to the cloud server before being displayed as a report within the tablet software. Our AI-based eye-tracking software utilizes a combination of computer vision techniques and deep learning architectures to estimate gaze direction and attention, a multi-stage pipeline likely incorporating the following components: face detection and landmark localization, head pose estimation, pupil center detection and tracking, and gaze estimation.

The demographic data (Name, Age, Date of birth, etc.) are entered into the user interface before the assessment begins. This data can be entered by the participant or by the assisting researcher. Participants’ distance was controlled before the task by using a head-pose calibration procedure. They were instructed to position their heads within a virtual frame displayed on the screen prior to the assessment, ensuring consistent distance and minimizing variations in apparent gaze position due to head movement. While we did not perform formal refractive corrections, participants were instructed to wear their usual corrective lenses. Additionally, we ensured that participants had clear visibility of the screen content before commencing the assessment.

### Test construction

**Fig. 1:**
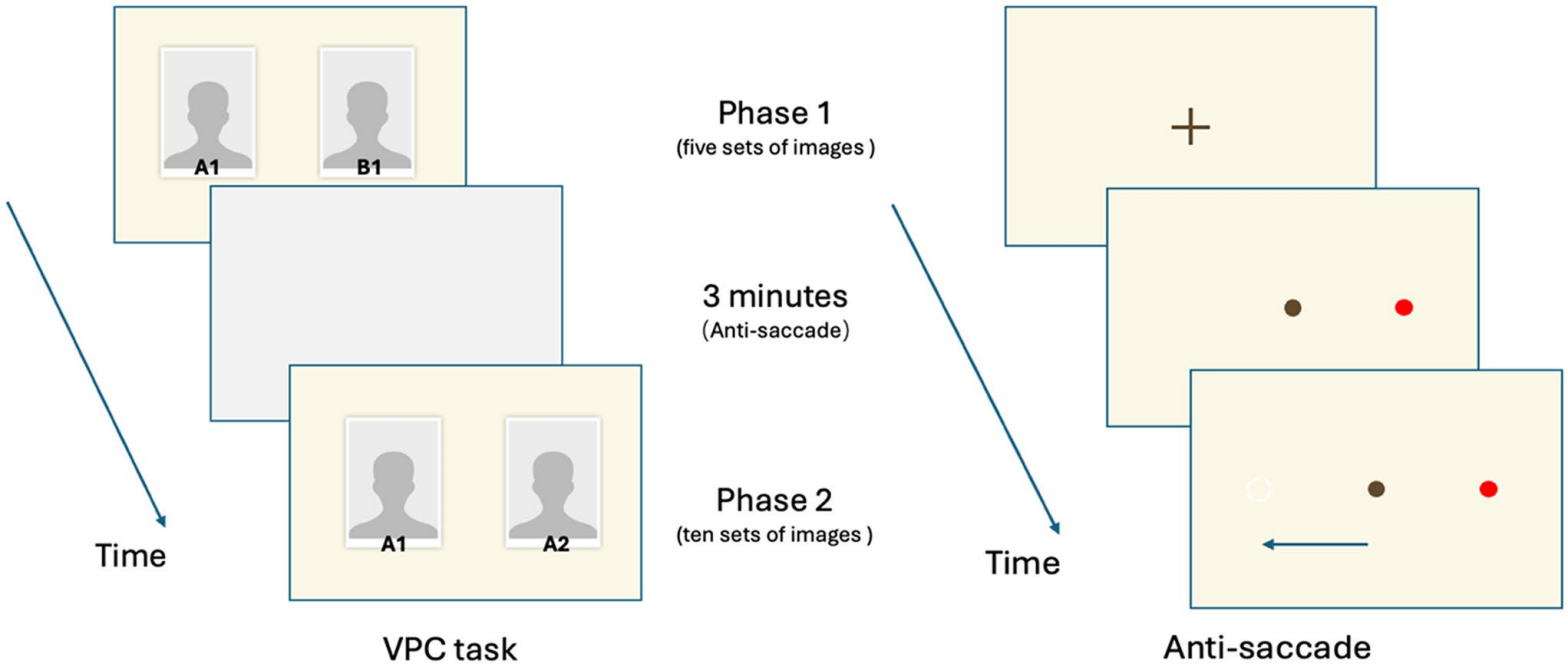
The VPC and antisaccade task

**Fig. 2:**
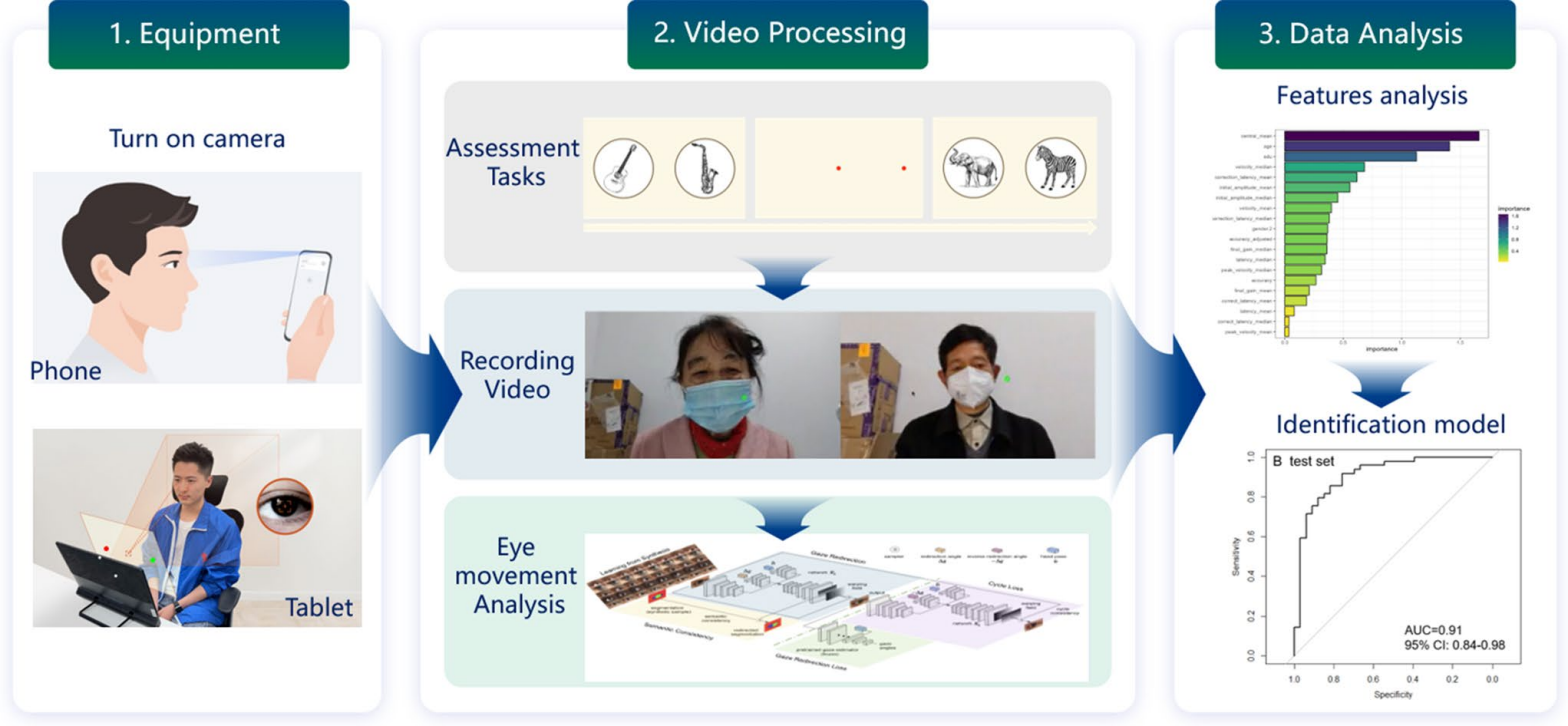
Scenery of the eye movement task

The total test duration is approximately 6 min, which consists of four parts: pre-task calibration, VPC task (Phase 1), anti-saccade task and VPC task (Phase 2). Pre-task calibration is used to calibrate the eye-tracking system by correcting the accuracy of model recognition on different types of devices, adjusting the position of the face in the camera frame, controlling the distance between the participant and the camera. In the first phase of VPC task, five sets of images were presented on the screen sequentially in random order. Participants were instructed to memorize these images and were informed that there would be a subsequent memory task related to these images. Each set of images was presented for 5 s. After the presentation of the five sets of images, an anti-saccade task was conducted (Fig. 1), lasting approximately 3 min. There are three variants of anti-saccade tasks, namely, gap, overlap, and immediate condition. The division of the three paradigms is based on the temporal difference between the disappearance of the central fixation point and the emergence of the target [32]. The overlap condition was used, in which the central fixation is still there when the target appears. Upon completion of the anti-saccade task, the second phase of the VPC task began. In this phase, ten sets of images were presented on the screen in random order. Each set of images consisted of one previously presented image (from the first phase) and one new image. Each set of images was presented for 6 s. Participants were instructed to look at the new image. During the VPC task, the tablet’s camera was only turned on during the second phase and recorded a video of the user’s eye movements during each trial. After processing the video, a series of eye movement-related parameters were established, as shown in Table 1. The scenery of the eye movement task, as well as the data collection and processing, are shown in Fig. 2.

### Eye movement parameters

**Table 1:** The parameters measured during the eye tracking test and their definitions

Parameters	Definition
Anti-saccade task	
Latency (median, ms)	Median time taken from the appearance of a target to the beginning of a saccade in response to a target
Latency (mean, ms)	Mean time taken from the appearance of a target to the beginning of a saccade in response to a target
Latency (correct median, ms)	Median time of correct reaction that taken from the appearance of a target to the beginning of a saccade in response to a target
Latency (correct mean, ms)	Mean time of correct reaction that taken from the appearance of a target to the beginning of a saccade in response to a target
Velocity (median, pixel/ms)	Median velocity of the saccade
Velocity (mean, pixel/ms)	Mean velocity of the saccade
Final gain (mean)	The ratio of the actual saccade amplitude to the distance between the final fixation point before the target appears and the target point.
Initial amplitude (median)	The median horizontal distance change of the first saccade after the target point appears.
Initial amplitude (mean)	The mean horizontal distance change of the first saccade after the target point appears.
Correction latency (median, ms)	The median latency for actively correcting an erroneous saccade: after the target appears, the direction of the first saccade is incorrect, but a subsequent saccade occurs in the correct direction. This latency is measured relative to the first saccade.
Correction latency (mean, ms)	The mean latency for actively correcting an erroneous saccade: after the target appears, the direction of the first saccade is incorrect, but a subsequent saccade occurs in the correct direction. This latency is measured relative to the first saccade.
Accuracy	Accuracy Percentage of accurately shifting eye gazes toward the stimuli in prosaccade trials and shift away from the stimuli in anti-saccade trials.
Adjusted accuracy	The sum of the percentage of successful initial shifts toward the eye gaze and the percentage of incorrectly directed initial saccades that were subsequently rectified in the correct direction within a trial.
VPC task	
central duration (median, ms)	The median time spent in the central area (± 100 ms from the center of the screen) during a simple fixation task

### Variables selection and development of machine learning model

As described above, 14 variables were collected from participants. However, not all of these eye movement parameters were deemed useful for constructing the machine learning model due to potential redundancy. Therefore, for the best performance of the model, selection of task-specific features is an essential procedure. For the Least absolute shrinkage and selection operator (LASSO) model [33–34], the L1 regularizer was used as the cost function, the error value of cross-validation is 10. After that, the remaining features were used to construct a machine learning algorithm. In this study, the training and test sets were randomly divided in a ratio of 2:8. Utilizing the selected optimal features, we employed Logistic Regression (LR), a machine learning classifier, for classification analysis. The test set was utilized to enhance the efficiency of these models. LR is a type of probabilistic statistical classification model that can be used to predict the classification of nominal variables based on certain features. The classification is accomplished by utilizing the logit function to evaluate the probability of outcomes.

### Statistical analysis

Statistical analyses were performed using the SPSS software 23.0 (IBM, NY) and R 4.3.1 software (http://www.Rproject.org). The Kolmogorov–Smirnov test was used to evaluate the normality of variable distributions. For data with a normal distribution, a t-test was used to evaluate the differences between groups. For data without a normal distribution, a Mann–Whitney test was used. For continuous variables with normality distribution, results were shown as the mean with standard deviation (SD). For variables without normality distribution, results were shown as the median with minimum and maximum. Categorical data were presented as frequencies, and the differences between groups were examined by Chi-square test. LASSO regression analysis was used for variables selection. By introducing the variables selected in the LASSO, we used multivariate logistic regression analysis to construct the prediction model. Backward step-wise selection was applied by using the likelihood ratio test with Akaike’s information criterion (AIC) as the stopping rule [35–36]. The variance inflation factor (VIF) was used to diagnose the collinearity of each variable, with VIF values greater than 10 indicated severe multicollinearity. The nomogram is based on proportionally converting each regression coefficient in logistic regression to a 0-100 points scale. For clinical use of the model, the total scores of each patient were calculated based on the nomogram. The predictive performance of the model was validated using the training and test sets, with diagnostic accuracy and calibration performance serving as evaluation metrics. These metrics were assessed through receiver operating characteristic (ROC) curve analysis and calibration curve analysis. Model calibration was tested using the Hosmer-Lemeshow test. Variable Importance in Projection (VIP) analysis was used for explain the importance of variables in prediction model.

## Results

### Demographics and eye movements features

The study included 273 participants with an average age of 70.46 ± 9.45 years, of whom 166 had AD and 107 were normal control subjects (Table 2). The age and education years in AD group [74 (50–90), 9 (6–18)] was significantly lower than that in NC group [67 (50–88), 12 (6–22)] (all with *P* < 0.001). Gender was not significantly different between the two groups. Cognitive assessments showed a significant decline of MMSE and MoCA-B in AD group [18 (0–24), 11 (0–20)] compared with MMSE and MoCA-B in NC [28 (22–30), 25 (18–30)] (all with *P* < 0.001). The latency (mean, ms), latency (correct mean, ms), and correction latency (mean, ms) in AD group were 345.25 (118.38-1069.64), 355.83 (237.11-1149.44), 172.36 (0-1444.29), respectively, which were significantly lower in NC group [278.48 (153.93-1087.88), 299.81 (259.63-1218.10), 133.32 (33.30-766.24)], all with *p* < 0.05. The final gain (mean) and central duration (median, ms) in AD group were 0.23 (0.05–18.89) and 1974.82 (1699.83-3799.66), which were significantly higher in NC group [0.30 (0.07–1.27) and 2736.26 (1919.26-4366.87)], with *p* < 0.005. All participants were randomly divided 8 to 2 into a training set and a test set, no significant differences were found between those two groups (Table 3).

**Table 2:** Demographical, cognitive performance scores and eye movements features of the participants

Variables *	Participants (*n* = 273)	NC (*n* = 107)	AD (*n* = 166)	*P* value
Age(years)	72 (50–90)	67 (50–88)	74 (50–90)	< 0.001
Sex (female)	167	69	98	0.220
Education years	9 (6–22)	12 (6–22)	9 (6–18)	< 0.001
MMSE	22 (0–30)	28 (22–30)	18 (0–24)	< 0.001
MoCA-B	14 (0–30)	25 (18–30)	11 (0–20)	< 0.001
latency (median, ms)	263.84 (112.66-1035.58)	244.52 (125.50-943.11)	276.24 (112.66-1035.58)	0.058
latency (mean, ms)	322.82 (118.38-1087.88)	278.48 (153.93-1087.88)	345.25 (118.38-1069.64)	< 0.001
latency (correct median, ms)	297.78 (197.65-1218.10)	276.14 (200.54-1218.10)	312.88 (197.65-1093.70)	0.109
latency (correct mean, ms)	321.43 (237.11-1218.10)	299.81 (259.63-1218.10)	355.83 (237.11-1149.44)	0.037
velocity (median, pixel/ms)	1.35 (0.81–3.61)	1.38 (0.87–3.61)	1.32 (0.81–2.88)	0.744
velocity (mean, pixel/ms)	1.41 (0.84–3.62)	1.41 (0.91–3.62)	1.41 (0.84–2.69)	0.579
final gain (mean)	0.25 (0.05–18.89)	0.30 (0.07–1.27)	0.23 (0.05–18.89)	0.003
initial amplitude (median)	12.10 (1.29–53.99)	12.61 (3.24–31.41)	11.99 (1.29–53.99)	0.643
initial amplitude (mean)	13.02 (4.73–57.42)	13.43 (4.87–30.49)	12.99 (4.73–57.42)	0.374
correction latency (median, ms)	117.85 (0-2033.10)	99.99 (33.30-666.59)	133.32 (0-2033.10)	0.155
correction latency (mean, ms)	149.98 (0-1444.29)	133.32 (33.30-766.24)	172.36 (0-1444.29)	0.010
accuracy (%)	0.43 (0-0.88)	0.44 (0-0.86)	0.43 (0-0.88)	0.158
adjusted accuracy (%)	0.80 (0.21-1.00)	0.90 (0.25-1.00)	0.71 (0.21-1.00)	< 0.001
central duration (median, ms)	2133.13 (1699.83-4366.87)	2736.26 (1919.26-4366.87)	1974.82 (1699.83-3799.66)	< 0.001

*: The Mann-Whitney test was employed to analyze continuous variables. Results were presented as median (min-max). ms: millisecond; %: percentage; MoCA-B, Montreal Cognitive Assessment Basic; MMSE, Minimum Mental State Examination; NC, normal control; AD, Alzheimer’s disease dementia. P value represents comparison between NC and AD

**Table 3:** Demographical, cognitive performance scores and eye movements features of participants in the training and test sets

Characteristic*	Training set (*n* = 218)	Test set (*n* = 55)	*P* value
NC/AD (n)	85/133	22/33	-
Age (years)	72 (50–90)	73 (50–88)	0.132
Sex (female)	137	30	0.165
Education years	9 (6–18)	12 (6–22)	0.151
MMSE	22 (0–30)	23 (4–30)	0.989
MoCA-B	14 (0–30)	15 (2–29)	0.925
latency (median, ms)	253.98 (125.50-1035.58)	270.96 (112.66-943.11)	0.053
latency (mean, ms)	315.87 (153.92-1069.64)	333.97 (118.38-1087.88)	0.056
latency (correct median, ms)	296.90 (197.65-1010.56)	305.17 (199.86-1218.10)	0.076
latency (correct mean, ms)	315.87 (253.92- 1069.64)	332.11 (237.11-1218.10)	0.080
velocity (median, pixel/ms)	1.39 (0.81–3.61)	1.24 (0.81–2.77)	0.055
velocity (mean, pixel/ms)	1.43 (0.86–3.62)	1.28 (0.84–2.99)	0.054
final gain (mean)	0.27 (0.05–18.89)	0.23 (0.06–1.12)	0.153
initial amplitude (median)	12.12 (3.24–53.99)	11.96 (1.29–25.58)	0.847
initial amplitude (mean)	13.02 (4.73)	13.17 (9.34–40.72)	0.936
correction latency (median, ms)	133.32 (0-747.08)	75.95 (33.30-2033.10)	0.848
correction latency (mean, ms)	155.53 (0-766.24)	137.60 (33.30-1444.29)	0.903
accuracy (%)	0.44 (0-0.88)	0.40 (0.11–0.82)	0.297
adjusted accuracy (%)	0.81 (0.21-1.00)	0.80 (0.25-1.00)	0.236
central duration (median, ms)	2133.09 (1699.83-4166.23)	2277.25 (1733.32-4366.87)	0.721

In training and test sets, the clinical characteristics were basically similar. *: The Mann-Whitney test was employed to analyze continuous variables. Results were presented as median (min-max)

### LASSO regression analysis and prediction model construction

**Fig. 3:**
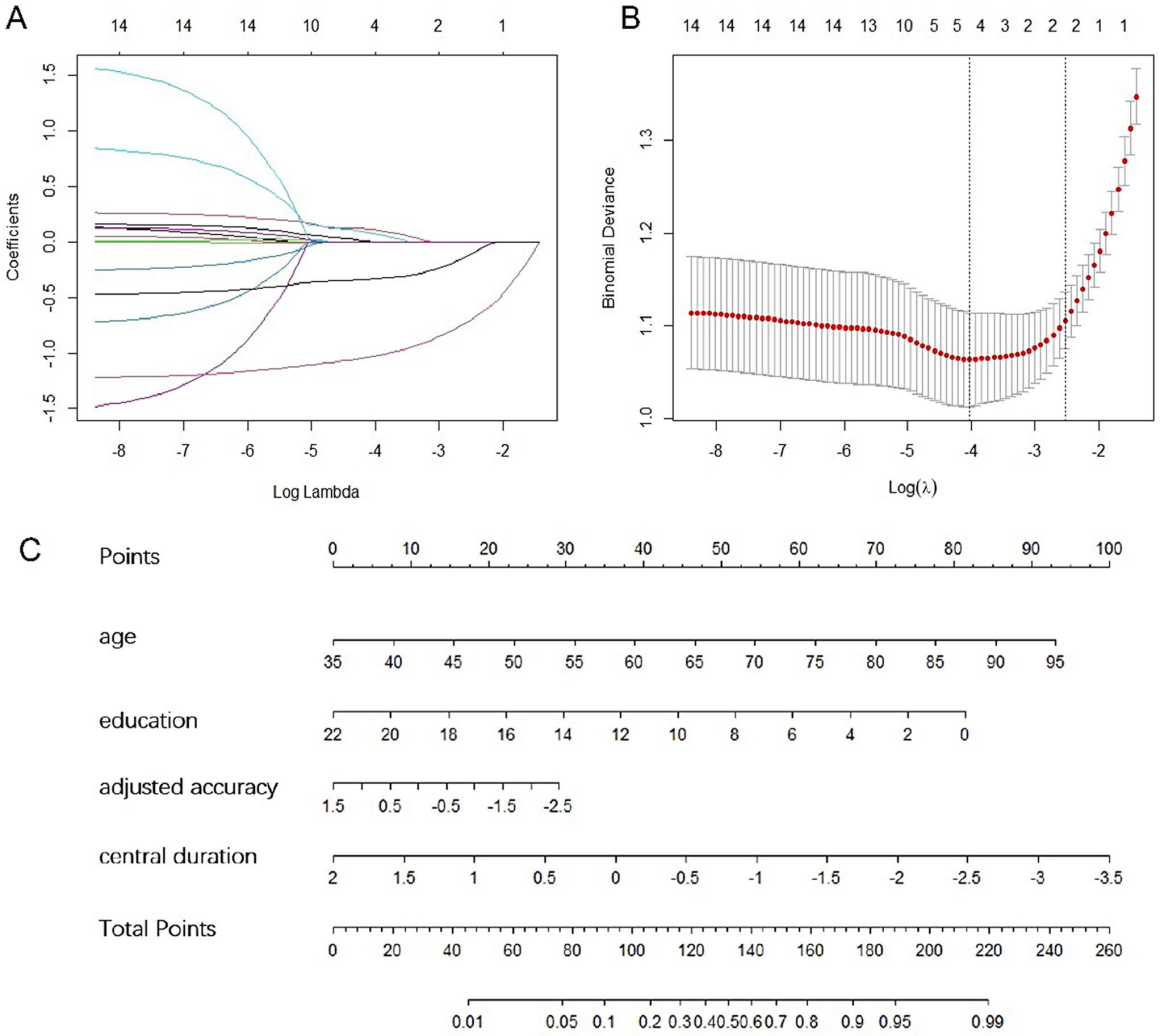
Features selection by least absolute shrinkage and selection operator (LASSO) and nomogram for the prediction model. (**A**) A coefficient profile plot was produced against the log lambda sequence. By deducing the best lambda, five variables with non-zero coefficients were selected; (**B**) Ten-fold cross-validation for tuning parameter selection in the LASSO model. The partial likelihood deviance (binomial deviance) curve was plotted versus log(λ). To avoid overfitting, 1 standard error was selected; (**C**) For example, by using the nomogram model, it could be concluded that a 60-year-old man, 10 years of education, with adjusted accuracy of -2.5, central duration of -2.5, had probably a 96% risk of AD

LASSO regression analysis was used to select the eye movements variables (Fig. 3A and B). 14 variables were reduced to 2 features, including adjusted accuracy and central duration (median, ms). Multivariate logistic analysis was further performed in the training set to establish the prediction model. Two of demographical variables, age and education years were included in the prediction model. Logistic regression analysis using backforward selection to test the independent variables, with age, education years, adjusted accuracy, and central duration included in the prediction model (Table 4). The inclusion of these variables led to the minimization of the AIC (209.88) in the prediction model. On multivariate analysis, with results reported as odds ratio (95% CI), age (1.09 [1.04, 1.14]), education years (0.82 [0.74, 0.92]), adjusted accuracy (0.68 [0.46, 1.00]), central duration (median, ms) (0.38 [0.25, 0.58]). Those four variables were introduced into the prediction model to develop the AD risk nomogram (Fig. 3C).

**Table 4:** Screening of predictors involved in the multivariate logistic regression

Variable	OR (95% CI)	*P* value	VIF value
Age	1.09 (1.04, 1.14)	< 0.001	1.15
Education years	0.82 (0.74, 0.92)	< 0.001	1.15
Adjusted accuracy	0.68 (0.46, 1.00)	< 0.05	1.07
Central duration (median, ms)	0.38 (0.25, 0.58)	< 0.001	1.07

OR, odd’s ratio; CI, confidence interval; VIF, variance inflation factor

### Prediction model verification

**Fig. 4:**
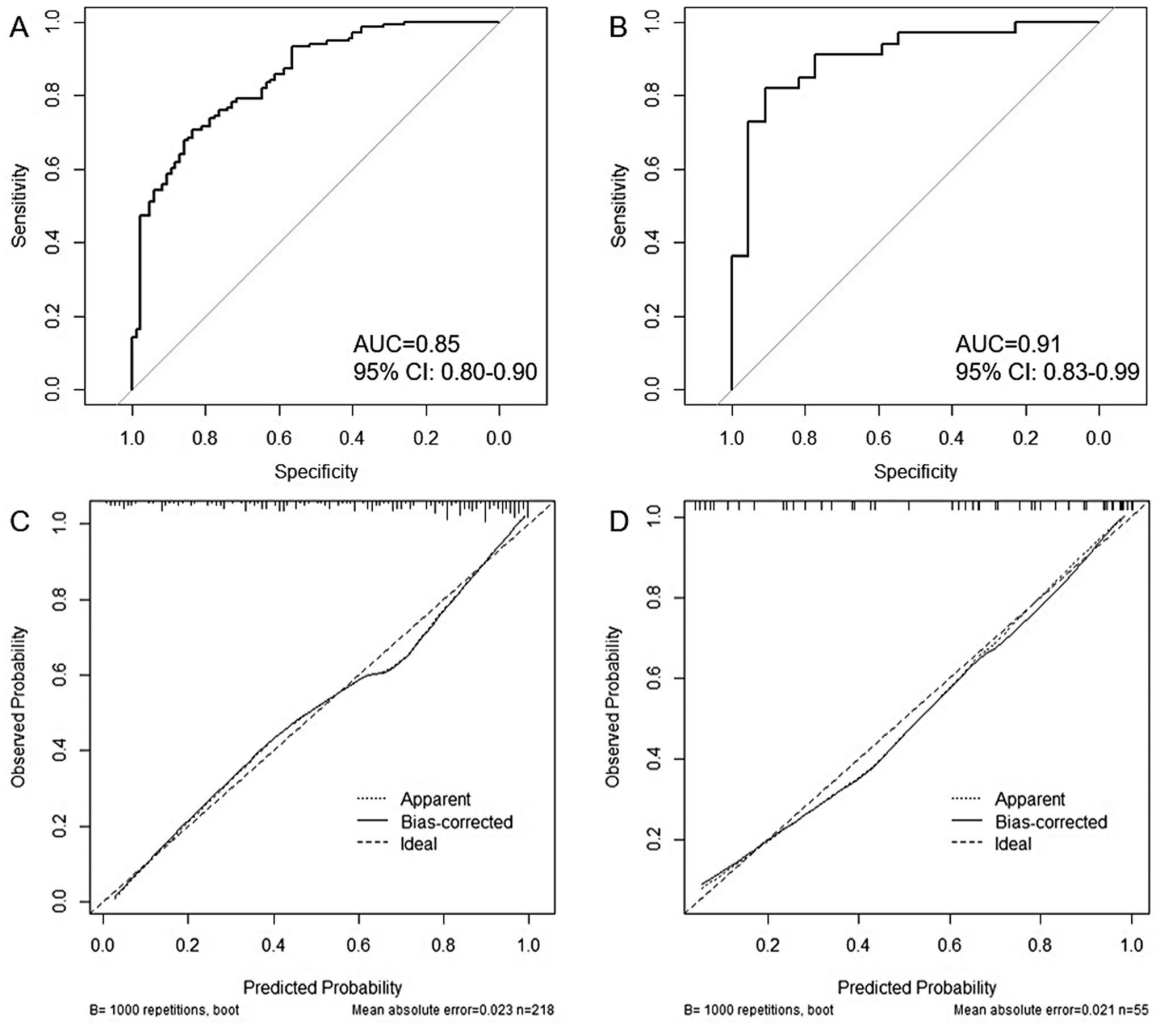
The ROC curve and calibration curve for the prediction model in training and test sets. (**A**): training set; (**B**): test set; (**C**): training set; (**D**): test set. Calibration curve: the black solid line above the x-axis represents sample distribution. The dotted lines on the diagonal represent the perfect prediction of the ideal model, and the solid lines represent the performance of the training set and the test set. The closer the solid line is to the dotted line, the better the predictive effect. The y-axis represents the actual diagnosed cases of AD, and the x-axis represents the predicted risk of AD

**Fig. 5:**
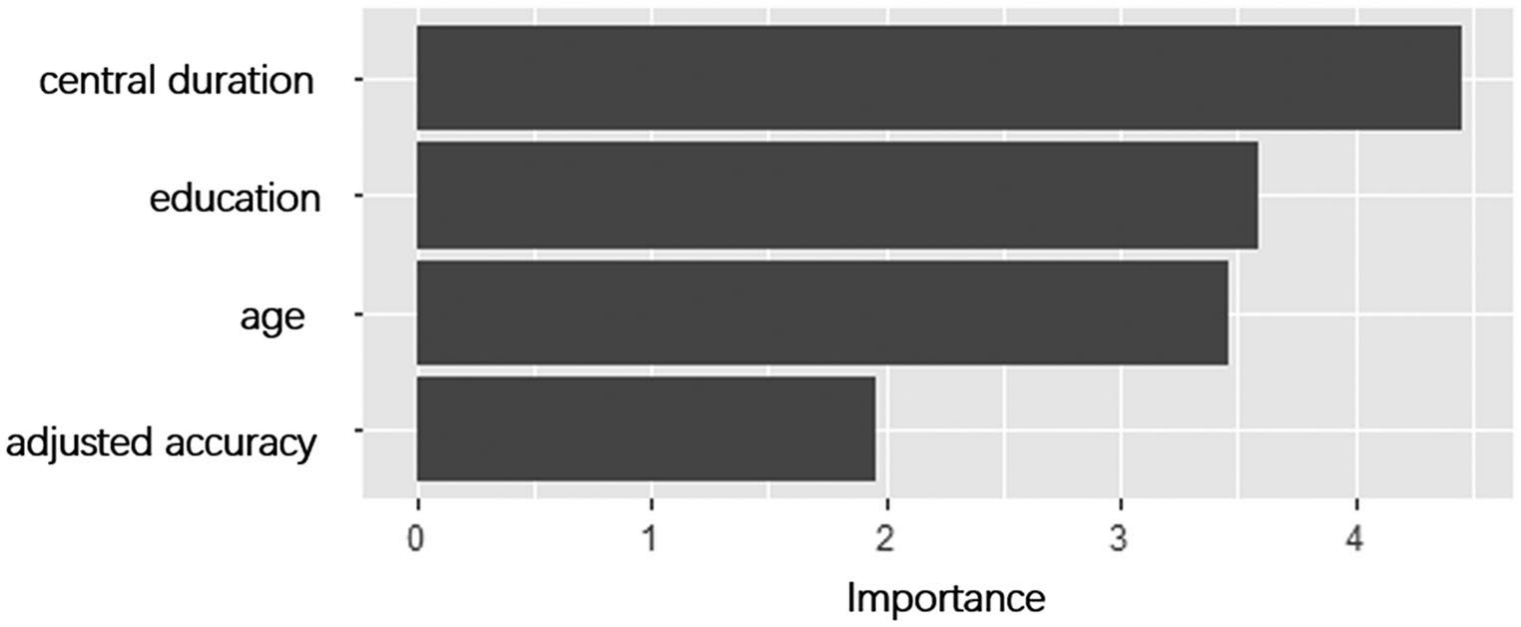
Variable importance plot for the logistic regression model

The ROC curve is used to assess the discriminating ability of the model (Fig. 4A and B). In the training set, the prediction model yielded an AUC of 0.85 (95%CI: 0.80, 0.90) to identify NC participants from AD (Table 5). The accuracy, sensitivity, specificity, positive predictive value (PPV), negative predictive value (NPV), positive likelihood ratio (PLR) and negative likelihood ratio (NLR) were 76%, 70.7%, 84%, 87%, 65%, 4.29, and 0.35 in the training set. In the test set, the model yielded an AUC of 0.91 (95%CI: 0.83, 0.99). The accuracy, sensitivity, specificity, PPV, NPV, PLR and NLR were 86%, 82%, 91%, 93%, 77%, 9.00, 0.20 in the test set. The variable importance plot (Fig. 5) implied that central duration, followed by education, age and adjusted accuracy are important features for the risk evaluation of AD for the logistic regression model.

Calibration curve (Fig. 4C and D) and Hosmer-Lemeshow test were used to calibrate the logistic regression model. The prediction model had a good fit with the test set. The Hosmer-Lemeshow test yielded a nonsignificant statistic in the training set and test set (*P* = 0.428 and *P* = 0.081, respectively), which suggested that there was no departure from perfect fit.

**Table 5:** Accuracy of the prediction of the nomogram

Variable	Value (95% CI)	
	Training set	Test set
Area under ROC curve	0.85 (0.80, 0.90)	0.91 (0.83, 0.99)
Accuracy	0.76 (0.76, 0.76)	0.86 (0.85, 0.86)
Sensitivity	0.71 (0.63, 0.78)	0.82 (0.69, 0.95)
Specificity	0.84 (0.76, 0.91)	0.91 (0.79, 1.00)
Positive predictive value	0.87 (0.81, 0.93)	0.93 (0.84, 1.02)
Negative predictive value	0.65 (0.56, 0.74)	0.77 (0.61, 0.93)
Positive likelihood ratio	4.29 (2.63, 7.01)	9.00 (2.38, 34.07)
Negative likelihood ratio	0.35 (0.27, 0.47)	0.20 (0.10, 0.42)

## Discussion

The purpose of this study is to assess the potential and usefulness of a novel eye-tracking task on mobile devices for evaluating eye movement parameters in both research and clinical practice settings. Furthermore, we developed a risk prediction model for AD dementia by combining eye movements and demographic factors. The study found that anti-saccade and VPC tasks variables, such as latency (mean, ms), correction latency (median, ms), correction latency (mean, ms), adjusted accuracy, and central duration (mean, ms) declined significantly in AD subjects compared with NC subjects. In multivariate logistic regression model, adjusted accuracy, central duration, age and education years are independent risk factors for AD. The model of eye movement variables combined with clinical demographical effectively discriminated AD and NC subjects. The AUC of 91%, sensitivity of 82%, and specificity of 91% obtained in the test set were comparable to previously published studies on AD and healthy subjects using eye movement parameters extracted with conventional equipment [20, 37]. The findings highlight the possibility of using eye-tracking technology for wider application of monitoring and early diagnosis.

Our eye movement findings are very much in line with those previously reported in scientific literature [38–40]. We found that the anti-saccade task scores, adjusted accuracy are decreased in AD. In the anti-saccade task, the most prevalent error occurs when participants move their gaze towards the displayed target instead of away from it. When participants shift their gaze towards the targets and make an initial error but quickly rectify it by diverting their attention away from the presented stimulus, this is referred to as a self-corrected error [41]. AD patients are prone to not correct mistakes due to alterations in the self-monitoring and correction network in brain, which is correlated with dysfunction of the prefrontal cortex and anterior cingulate region [41].

The eye movement latency refers to the time interval between the appearance of a stimulus and the initiation of a saccadic response. When programming a saccadic eye movement, a decision-making process takes place prior to the eye movement. The initiation of a saccadic eye movement relies on the resources of executive function and attentional processing capabilities. Greater latencies could be found in subjects with AD, which may indicate executive function impairment and attentional deficits [39]. In our study, we found that the latency scores, including correction latency and latency (mean, ms) are increased in AD. This result aligns with previous studies, the percentage of the anti-saccade latency of patients with AD was higher than healthy control [38–40]. In our study, we observed a decrease in the central duration among AD subjects, which is in line with previous study [8, 41–42]. VPC tasks could quantify how the participant split attention between familiar and novel visual stimuli. Healthy subjects spend a greater proportion of time gazing at the novel images than the familiar images in the VPC task. Individuals with AD have trouble to remember previously viewed images and prone to spend an equal amount of time and attention gazing at both novel and previously viewed images [41–42].

Traditionally, commercial-grade eye-trackers have been used for eye movement tests, employing high frame-rate cameras capable of capturing various intricate visual features [16, 37]. Those trackers are a common method for tracing eye movements, and have been validated to be highly effective in research and clinical settings [16, 37, 41]. However, those equipment is quite expensive and not easily accessible, which limits its widespread use and evaluation. The ability to utilize the built-in cameras of smart devices allows us to overcome these cost and scalability barriers by democratizing access to eye-tracking assessment tools. Bott and Gills have validated the use of web cameras on computer or smart devices for VPC tasks as an alternative to the traditional eye-tracking equipment to assess cognitive function [18, 43]. The AUC (area under the curve) of 0.80 for VPC scores demonstrated good classification accuracy for MCI and cognitively normal control [43].

Classification algorithms for predictive models have increasingly been adapted in medical research, particularly within the field of neurology, to facilitate the detection of neurodegenerative conditions [16, 44]. Machine learning combined with eye-tracking technologies have also shown good performance in detecting cognitive impairments in AD patients [16, 45–46]. In a recent study, Fangyu Zuo and colleagues used a multilayered comparison convolutional neural network (MC-CNN) based on eye-tracking data to distinguish AD patients from normal controls with a accuracy of 0.84 and AUC of 0.90 [46]. In our study, LR model based on eye movements data and demographics demonstrate a good classification accuracy for AD and cognitively normal controls, which with an AUC of 0.91 in test set. The entire process has been proven effective for diagnosing AD on the current dataset. The logistic regression model possesses the advantage of being interpretable. Higher age and lower level of education years, adjusted accuracy, and central duration are the risk factors for developing AD in the model. As mentioned above, adjusted accuracy and central duration are typically decreased in AD subjects [41–42]. Ageing is accompanied by changes in the brain, including general atrophy and an imbalance in amyloid-β production and degradation [47], which is the most common risk factor in AD [40]. Low education is another common risk factor for AD, higher level of education might increase the brain’s cognition [48].

An advantage of eye-tracking monitoring technologies is that they have the potential to be more easily scaled to neurodegenerative disorders. Several eye-movement anomalies have been shown to highly correlate with Parkinson’s Disease [24] and multiple sclerosis [49]. Moreover, the eye movement task requires minimal instruction by examiner. Only a brief instruction regarding the procedure during the assessment, with text subtitles on screen and voice-overs, making it accessible to illiterate individuals. Another strength of this study is the efficient and convenient use of mobile devices to collect eye movement data, making it accessible for AD patients to complete task in primary clinical settings or follow up at home. This non-invasive and easily accessible method may have many applications which can help identification of AD patients, enrollment of AD drug clinical trials and assessment of AD treatments.

To conclude, this study demonstrates the potential utility of a novel mobile eye-tracking technology in identifying differences in eye movement abnormalities associated with AD. Moreover, based on the collected eye movement data, we were able to effectively differentiate individuals with AD from healthy participants with high sensitivity. However, there are still several disadvantages that need to be addressed. One of the main drawbacks is that the integration of data collection and analysis was not fully implemented. The eye movement paradigm still needs to be validated in a larger sample size to enhance the robustness of the results; therefore, currently, these two processes are carried out separately. Additionally, AD represents a continuum of diverse cognitive stages; future studies should incorporate multiple cognitive cohorts to construct a multi-classification prediction model. Moreover, the app currently only supports Android devices, which restricts its usability on different operating systems. Improving compatibility with various operating systems will be a key focus for future development. This study is part of an ongoing large-scale multi-center eye movement study and future investigations will assess the predictive capacity in different cognitive stages and incorporate other modalities such as molecular imaging and blood based biomarkers. Furthermore, in the future, we will integrate an application with more optimized algorithm models, such as deep learning models that combine eye-tracking and diagnosis procedure to facilitate clinical utilization.

## Conclusion

In this study, we employed AI-driven eye-tracking technology on a tablet to collect eye movement parameters and identified significant differences in multiple eye movement parameters between individuals with AD and healthy controls. Subsequently, we developed a prediction model that integrated distinctive eye-tracking features with demographic information, which demonstrated good efficacy in classification. These findings suggest the potential of utilizing eye-tracking technology for screening of AD patients, thereby highlighting its broader applications in monitoring and early diagnosis.

## Data Availability

No datasets were generated or analysed during the current study.
